# Supplementary material for: Plasma membrane overgrowth causes fibrotic collagen accumulation and immune activation in Drosophila adipocytes
Source: eLife. 2015 Jun 19;4:e07187. doi: 10.7554/eLife.07187 (PMC4490375; doi:10.7554/eLife.07187)
Supplement: Supplementary file 1. — Hits in RNAi screening causing plasma membrane accumulation of Collagen IV in adipocytes. DOI: http://dx.doi.org/10.7554/eLife.07187.019 [file elife07187s001.docx]

## Supplementary File 1

Hits in RNAi screening causing plasma membrane accumulation of Collagen IV in adipocytes.

| **TRIP ID** | **THU ID** | **CG number** | **Symbol** | **Full name** | **Category** | **Melanization** |
| --- | --- | --- | --- | --- | --- | --- |
| HMS00154 | THU0685 | *CG18102* | *shi* | *shibire* | Endocytosis | ++ |
| JF03133 | THU5901 | *CG18102* | *shi* | *shibire* | Endocytosis | ++ |
| HMC03420 | TH02192.N | *CG3664* | *Rab5* | *Rab5* | Endocytosis | ++ |
| HMS00147 | THU0679 | *CG3664* | *Rab5* | *Rab5* | Endocytosis | ++ |
| JF03085 | THU3062 | *CG8085* | *RN-tre* | *Related to the N terminus of tre oncogene* | Endocytosis | ++ |
| JF02963 | THU2965 | *CG12532* | *AP-1-2β* | *Adaptor Protein complex 1/2, β subunit* | Endocytosis | ++ |
| HMS00653 | THU1055 | *CG4260* | *AP-2α* | *Adaptor Protein complex 2, α subunit* | Endocytosis | ++ |
| JF02875 | THU2879 | *CG7057* | *AP-2μ* | *Adaptor Protein complex 2, μ subunit* | Endocytosis | ++ |
| JF02681 | THU2693 | *CG9012* | *Chc* | *Clathrin heavy chain* | Endocytosis | - * |
| HMS00841 | THU1233 | *CG2903* | *Hrs* | *Hepatocyte growth factor regulated tyrosine kinase substrate* | Endocytosis | - |
| JF02860 | THU2864 | *CG2903* | *Hrs* | *Hepatocyte growth factor regulated tyrosine kinase substrate* | Endocytosis | - |
| HMS00084 | THU0621 | *CG5848* | *cact* | *cactus* | Signaling | ++ |
| GL00627 | THU4526 | *CG5848* | *cact* | *cactus* | Signaling | ++ |
| GL00021 | THU0020 | *CG2028* | *CkIα* | *Casein kinase Iα* | Signaling | ++ |
| JF01792 | THU5828 | *CG2028* | *CkIα* | *Casein kinase Iα* | Signaling | - |
| HMS00161 | THU0692 | *CG17998* | *Gprk2* | *G protein-coupled receptor kinase 2* | Signaling | + |
| HMS00037 | THU0575 | *CG15793* | *Dsor1* | *Downstream of raf1* | Signaling | - |
| JF01295 | THU1921 | *CG4303* | *Bap60* | *Brahma associated protein 60kD* | Chromatin | ++ |
| JF02413 | THU2456 | *CG4013* | *Smr* | *Smarter* | Chromatin | ++ |
| GL00488 | THU4498 | *CG32443* | *Pc* | *Polycomb* | Chromatin | ++ |
| GL00477 | THU4495 | *CG18740* | *mor* | *moira* | Chromatin | ++ |
| HMS01267 | THU3717 | *CG18740* | *mor* | *moira* | Chromatin | + |
| HMS00167 | THU0698 | *CG33554* | *Nipped-A* | *Nipped-A* | Chromatin | + |
| HMS00050 | THU0588 | *CG5942* | *brm* | *brahma* | Chromatin | ++ |
| HM04019 | THU4855 | *CG5942* | *brm* | *brahma* | Chromatin | - |
| GL00255 | THU0229 | *-* | *His3* | *Histone 3* | Chromatin | + |
| HMS00450 | THU0898 | *CG7098* | *dik* | *diskette* | Chromatin | - |
| HMS00363 | THU0824 | *CG1064* | *Snr1* | *Snf5-related 1* | Chromatin | - |
| HMJ03128 | THU4981 | *CG2128* | *Hdac3* | *Histone deacetylase 3* | Chromatin | - |
| HMS02564 | THU5431 | *CG4677* | *lmd* | *lame duck* | Chromatin | - |
| HMS01911 | THU3913 | *CG14542* | *Vps2* | *Vacuolar protein sorting 2* | Other traffic | + |
| JF02684 | THU2696 | *CG9113* | *AP-1γ* | *Adaptor Protein complex 1, γ subunit* | Other traffic | - |
| HMS01349 | THU1574 | *CG7053* | *Atg101* | *Autophagy-related 101* | Other traffic | - |
| JF02786 | THU2792 | *CG1241* | *Atg2* | *Autophagy-related 2* | Other traffic | - |
| HMS02647 | THU4466 | *CG5075* | *Vha68-3* | *Vacuolar H^+^ ATPase 68kD subunit 3* | Other traffic | - |
| GL01171 | THU4608 | *CG3157* | *γTub23C* | *γ−Tubulin 23C* | Cytoskeleton | ++ |
| JF01373 | THU4011 | *CG1913* | *αTub84B* | *α-Tubulin 84B* | Cytoskeleton | + |
| GL00253 | THU0227 | *CG8201* | *par-1* | *par-1* | Cytoskeleton | + |
| HMS00405 | THU0856 | *CG8201* | *par-1* | *par-1* | Cytoskeleton | - |
| HMC02992 | TH01477.N | *CG12051* | *Act42A* | *Actin 42A* | Cytoskeleton | - |
| HMJ03120 | THU4973 | *CG4347* | *UGP* | *UTP:glucose-1-phosphate uridylyltransferase* | Glucid metabolism | - |
| - | TH02425.N | *CG4347* | *UGP* | *UTP:glucose-1-phosphate uridylyltransferase* | Glucid metabolism | - |
| HMC03367 | TH02222.N | *CG17645* | *Pglym87* | *Phosphoglycerate mutase at 87* | Glucid metabolism | - |
| HMS02947 | THU5684 | *CG10166* | *-* | *-* | Glucid metabolism | - |
| HMS00779 | THU1172 | *CG2720* | *Hop* | *Hsp70/Hsp90 organizing protein homolog* | Chaperone | - |
| HMS00899 | THU1286 | *CG1242* | *Hsp83* | *Heat shock protein 83* | Chaperone | - |
| HMS00639 | THU1041 | *CG5374* | *T-cp1* | *Tcp1-like* | Chaperone | - |
| HMS00634 | THU1036 | *CG9193* | *PCNA* | *Proliferating cell nuclear antigen* | Other | ++ |
| - | TH03713.N | *CG14030* | *Bub1* | *Bub1 homologue* | Other | ++ |
| HMS02566 | THU4417 | *CG13298* | *-* | *-* | Other | ++ |
| GL01530 | THU4730 | *CG2682* | *d4* | *d4* | Other | ++ |
| HMS00242 | THU0736 | *CG18332* | *CSN3* | *COP9 signalosome subunit 3* | Other | + |
| HM05052 | THU0423 | *CG4916* | *me31B* | *maternal expression at 31B* | Other | + |
| HMS00685 | THU1086 | *CG12372* | *spt4* | *spt4* | Other | + |
| HMC02379 | TH01618.N | *CG10920* | *-* | *-* | Other | + |
| HMS02133 | THU5266 | *CG6378* | *BM-40-SPARC* | *BM-40-SPARC* | Other | - |
| JF02666 | THU2678 | *CG42670* | *ps* | *pasilla* | Other | - |
| - | TH02920.N | *CG42670* | *ps* | *pasilla* | Other | - |
| HMS00537 | THU0975 | *CG1462* | *Aph-4* | *Alkaline phosphatase 4* | Other | - |
| HMS00195 | THU0712 | *CG7380* | *baf* | *barrier to autointegration factor* | Other | - |
| JF02257 | THU2343 | *CG8127* | *Eip75B* | *Ecdysone-induced protein 75B* | Other | - |
| HMC02435 | TH01755.N | *CG18331* | *Muc68Ca* | *Mucin 68Ca* | Other | - |
| HMC03457 | TH02343.N | *CG6385* | *Sardh* | *Sarcosine dehydrogenase* | Other | - |
| HM05087 | THU3482 | *CG9326* | *vari* | *varicose* | Other | - |
| HM05118 | THU0455 | *CG3413* | *wdp* | *windpipe* | Other | - |
| HMS02856 | THU5654 | *CG5266* | *Pros25* | *Proteasome α2 subunit* | Other | - |
| - | TH02984.N | *CG32604* | *l(1)G0007* | *lethal (1) G0007* | Other | - |
| GL01163 | THU4602 | *CG1812* | *-* | *-* | Other | - |
| JF02252 | THU2338 | *CG2116* | *-* | *-* | Other | - |
| JF02939 | THU2943 | *CG32204* | *-* | *-* | Other | - |

++ Melanization at 25^0^C + Melanization at 30^0^C * *Chc^DN^* shows melanization (++)
