# Supplementary material for: Plasma membrane overgrowth causes fibrotic collagen accumulation and immune activation in Drosophila adipocytes
Source: eLife. 2015 Jun 19;4:e07187. doi: 10.7554/eLife.07187 (PMC4490375; doi:10.7554/eLife.07187)
Supplement: Supplementary file 2. — Detailed genotypes. DOI: http://dx.doi.org/10.7554/eLife.07187.020 [file elife07187s002.docx]

## Supplementary File 2

Detailed genotypes.

| **Figure 1** |  |
| --- | --- |
| B | *w / +* or *Y ; vkg^G454^ UAS-myr.RFP/+ ; BM-40-SPARC-GAL4 UAS-Dcr2* / +  *w / y sc v* or *Y ; vkg^G454^ UAS-myr.RFP/+ ; BM-40-SPARC-GAL4 UAS-Dcr2* / *UAS-shi.RNAi^TRiP.JF03133^*  *w ; vkg^G454^ UAS-myr.RFP /* *UAS-shi.K44A ; BM-40-SPARC-GAL4 UAS-Dcr2* / + |
| C | *Canton-S*  *y w / y sc v* or *Y ; BM-40-SPARC-GAL4* / *UAS-shi.RNAi^TRiP.JF03133^*  *y w ; UAS-shi.K44A / + ; BM-40-SPARC-GAL4 / +* |
| D | *w ; vkg^G454^ UAS-myr.RFP/+ ; BM-40-SPARC-GAL4 UAS-Dcr2* / +  *w / y sc v* or *Y ; vkg^G454^ UAS-myr.RFP / + ; BM-40-SPARC-GAL4 UAS-Dcr2* / *UAS-shi.RNAi^TRiP.JF03133^* |
| E | *w / y sc v* or *Y; vkg^G454^ UAS-myr-RFP / UAS-Rab5.RNAi^TRiP.HMC03420^; BM-40-SPARC-GAL4 UAS-Dcr2* / +  *w ; vkg^G454^ UAS-myr-RFP / + ; BM-40-SPARC-GAL4 UAS-Dcr2* / *UAS-Rab5.S43N* |
| F | *w / y sc v* or *Y; vkg^G454^ UAS-myr-RFP/ UAS-Chc.RNAi^TRIP.JF02681^ ; BM-40-SPARC-GAL4 UAS-Dcr2/+*  *w ; vkg^G454^ UAS-myr-RFP/+ ; BM-40-SPARC-GAL4 UAS-Dcr2 / UAS-Chc.DN* |
| G | *w / +* or *Y ; vkg^G454^ UAS-myr.RFP / + ; BM-40-SPARC-GAL4 UAS-Dcr2* / +  *w / y sc v* or *Y; vkg^G454^ UAS-myr-RFP / + ; BM-40-SPARC-GAL4 UAS-Dcr2* / *UAS-shi.RNAi^TRiP.JF03133^*  *w ; vkg^G454^ UAS-myr-RFP /* *UAS-shi.K44A ; BM-40-SPARC-GAL4 UAS-Dcr2* / +  *w / y sc v* or *Y; vkg^G454^ UAS-myr-RFP/ UAS-Rab5.RNAi^TRiP.HMC03420^; BM-40-SPARC-GAL4 UAS-Dcr2* / +  *w ; vkg^G454^ UAS-myr-RFP / + ; BM-40-SPARC-GAL4 UAS-Dcr2* / *UAS-Rab5.S43N*  *w / y sc v* or *Y; vkg^G454^ UAS-myr-RFP / UAS-Chc.RNAi^TRIP.JF02681^ ; BM-40-SPARC-GAL4 UAS-Dcr2 / +*  *w ; vkg^G454^ UAS-myr-RFP / + ; BM-40-SPARC-GAL4 UAS-Dcr2 / UAS-Chc.DN* |
| H | *w / +* or *Y ; vkg^G454^ UAS-myr-RFP/+ ; BM-40-SPARC-GAL4 UAS-Dcr2* / +  *w ; vkg^G454^ UAS-myr-RFP /* *UAS-shi.K44A ; BM-40-SPARC-GAL4 UAS-Dcr2* / +  *w ; vkg^G454^ UAS-myr-RFP / + ; BM-40-SPARC-GAL4 UAS-Dcr2* / *UAS-Rab5.S43N* |
| **Figure 2** |  |
| A | *w / y sc v* or *Y; vkg^G454^ UAS-myr.RFP / + ; BM-40-SPARC-GAL4 UAS-Dcr2* / *UAS-shi.RNAi^TRiP.JF03133^* |
| B-D | *w / y sc v* or *Y ; vkg^G454^ / + ; BM-40-SPARC-GAL4 UAS-Dcr2* / *UAS-shi.RNAi^TRiP.JF03133^* |
| E | *w ; vkg^G454^ UAS-myr-RFP / + ; BM-40-SPARC-GAL4 UAS-Dcr2 / +*  *w / y sc v* or *Y; vkg^G454^ / + ; BM-40-SPARC-GAL4 UAS-Dcr2* / *UAS-shi.RNAi^TRiP.JF03133^*  *w / y sc v* or *Y ; vkg^G454^ / + ; BM-40-SPARC-GAL4 UAS-Dcr2* / *UAS-Tango1.RNAi^VDRC.v21594^* |
| F | *y w hs-Flp1.22 / y sc v* or *Y ; vkg^G454^ act-y+-GAL4 UAS-myr.RFP / + ; UAS-shi.RNAi^TRiP.JF03133^ / +*  *y w hs-Flp1.22 / y sc v* or *Y; vkg^G454^ act-y+-GAL4 UAS-myr.RFP / + ; UAS-shi.RNAi^TRiP.JF03133^ / UAS-GFP.dsRNA* |
| G | *y w hs-Flp1.22 / y sc v* or *Y ; act-y+-GAL4 UAS-GFP / UAS-Cg25C-RFP.2.1 ; UAS-shi.RNAi^TRiP.JF03133^ / +* |
| H | *w ; Cg-GAL4 UAS-myr.RFP / + ; UAS-hTfR.GFP / +* |
| I | *w / y sc v* or *Y; Cg-GAL4 UAS-myr.RFP / + ; UAS-hTfR.GFP / UAS-shi.RNAi^TRiP.JF03133^* |
| J | *w / y sc v* or *Y; Cg-GAL4 UAS-myr.RFP / + ; UAS-hTfR.GFP / UAS-Rab11.RNAi^TRIP.JF02812^* |
| **Figure 3** |  |
| A | *w ; BM-40-SPARC-GAL4 UAS-myr.RFP / TM6B*  *w / y sc v* or *Y ; BM-40-SPARC-GAL4 UAS-myr.RFP* / *UAS-shi.RNAi^TRiP.JF03133^*  *w / y sc v* or *Y ; UAS-Rab5.RNAi^TRiP.HMC03420^ / + ; BM-40-SPARC-GAL4 UAS-myr.RFP* / +  *w ; UAS-Chc.RNAi^VDRC.v244789^ / + ; BM-40-SPARC-GAL4 UAS-myr.RFP* / +  *w / y sc v* or *Y ; BM-40-SPARC-GAL4 UAS-myr.RFP* / *UAS-Rab11.RNAi^TRIP.JF02812^* |
| B | *y w / + ; BM-40-SPARC-GAL4* / +  *y w / y sc v* or *Y ; BM-40-SPARC-GAL4* / *UAS-shi.RNAi^TRiP.JF03133^*  *y w / y sc v* or *Y ; UAS-Rab5.RNAi^TRiP.HMC03420^ / + ; BM-40-SPARC-GAL4* / +  *w ; UAS-Chc.RNAi^VDRC.v244789^ / + ; BM-40-SPARC-GAL4* / +  *y w / y sc v* or *Y ; BM-40-SPARC-GAL4* / *UAS-Rab11.RNAi^TRIP.JF02812^* |
| C | *w ; BM-40-SPARC-GAL4 UAS-myr.RFP / TM6B*  *w / y sc v* or *Y ; BM-40-SPARC-GAL4 UAS-myr.RFP* / *UAS-Rab11.RNAi^TRIP.JF02812^*  *w / y sc v* or *Y; BM-40-SPARC-GAL4 UAS-myr.RFP* / *UAS-shi.RNAi^TRiP.JF03133^*  *w ;* *UAS-shi.K44A / + ; BM40-SPARC-GAL4 UAS-myr.RFP /+*  *w / y sc v* or *Y ; UAS-Rab5.RNAi^TRiP.HMC03420^ / + ; BM-40-SPARC-GAL4 UAS-myr.RFP* / +  *w ; BM-40-SPARC-GAL4 myr.RFP* / *UAS-Rab5.S43N*  *w / y v sc* or *Y ; UAS-Chc.RNAi^VDRC.v244789^ / + ; BM-40-SPARC-GAL4 UAS-myr.RFP* / +  *w ; BM-40-SPARC-GAL4 UAS-myr.RFP* */ UAS-Chc.DN*  *y w / + ; BM40-SPARC-GAL4* / +  *y w / y sc v* or *Y ; BM-40-SPARC-GAL4* / *UAS-Rab11.RNAi^TRIP.JF02812^*  *y w / y sc v* or *Y ; BM40-SPARC-GAL4* / *UAS-shi.RNAi^TRiP.JF03133^*  *y w / y sc v* or *Y ; UAS-Rab5.RNAi^TRiP.HMC03420^ / + ; BM-40-SPARC-GAL4* / +  *w ; UAS-Chc.RNAi^VDRC.v244789^ / + ; BM-40-SPARC-GAL4* / + |
| D | *w / +* or *Y ; vkg^G454^ UAS-myr.RFP/+ ; BM-40-SPARC-GAL4 UAS-Dcr2* / +  *w / y sc v* or *Y ; vkg^G454^ UAS-myr.RFP/+ ; BM-40-SPARC-GAL4 UAS-Dcr2* / *UAS-shi.RNAi^TRiP.JF03133^* |
| **Figure 4** |  |
| B | *w trol^CPTI-002049^ / Y*  *w trol^CPTI-002049^ / Y; BM40-SPARC-GAL4 UAS-myr.RFP / UAS-EGFP.shRNA.3* |
| **Figure 5** |  |
| A | *w trol^CPTI-002049^ / Y ; BM40-SPARC-GAL4 UAS-myr.RFP / TM2*  *w trol^CPTI-002049^ / Y ; BM40-SPARC-GAL4 UAS-myr.RFP / UAS-shi.RNAi^TRiP.JF03133^* |
| B | *w trol^CPTI-002049^ / Y ; UAS-shi.K44A / + ; BM40-SPARC-GAL4 UAS-myr.RFP /+*  *w trol^CPTI-002049^ / Y ; UAS-Rab5.RNAi^TRiP.HMC03420^ / + ; BM40-SPARC-GAL4 UAS-myr.RFP / +* |
| C, D | *w trol^CPTI-002049^ / Y ; UAS-shi.RNAi^NIG.18102R-1^ / + ; r4-GAL4 UAS-myr.RFP / +*  *w trol^CPTI-002049^ / Y ; UAS-shi.RNAi^NIG.18102R-1^ / + ; r4-GAL4 UAS-myr.RFP / UAS-vkg. RNAi^NIG.16858R-3^ UAS-Cg25C.RNAi^VDRC.v28369^* |
| E | *w / y sc v* or *Y ; UAS-myr.RFP vkg^G454^ / UAS-shi.RNAi^NIG.18102R-1^ ; BM40-SPARC-GAL4 UAS-Dcr2 / +*  *w / y sc v* or *Y ; UAS-myr.RFP vkg^G454^ / UAS-shi.RNAi^NIG.18102R-1^ ; BM-40-SPARC-GAL4 UAS-Dcr2 / UAS-PH4αEFB.RNAi^VDRC.v2464^* |
| F | *w / y sc v* or *Y ; UAS-secr.GFP / + ; BM-40-SPARC-GAL4 UAS-myr.RFP* / *UAS-shi.RNAi^TRiP.JF03133^*  *w / y sc v* or *Y ; UAS-secr.GFP / + ; BM-40-SPARC-GAL4 UAS-myr.RFP* / *UAS-Rab1.RNAi^TRiP^* *^JF02609^* |
| **Figure 6** |  |
| A | *w / y sc v* or *Y ; UAS-cact.RNAi^TRiP.GL00627^/ vkg^G454^ UAS-myr.RFP; BM-40-SPARC-GAL4 UAS-Dcr2/+* |
| B | *w ; UAS-Tl^10B^ / vkg^G454^ UAS-myr.RFP ; BM-40-SPARC-GAL4 UAS-Dcr2 / +* |
| C | *w ; vkg^G454^ UAS-myr.RFP / + ; BM-40-SPARC-GAL4 UAS-Dcr2 / +* |
| D | *w / y sc v* or *Y ; UAS-cact.RNAi^TRiP.GL00627^ / + ; BM-40-SPARC-GAL4 UAS-Dcr2 / +*  *w ; UAS-Tl^10B^ / + ; BM-40-SPARC-GAL4 UAS-Dcr2 / +* |
| E | *y w / + ; BM-40-SPARC-GAL4* / +  *w / y sc v* or *Y ; UAS-cact.RNAi^TRiP.GL00627^ / + ; BM-40-SPARC-GAL4 UAS-Dcr2 / +*  *w ; UAS-Tl^10B^ / + ; BM-40-SPARC-GAL4 UAS-Dcr2 / +*  *w ; BM-40-SPARC-GAL4 UAS-myr.RFP / +*  *w / y sc v* or *Y ; UAS-cact.RNAi^TRiP.GL00627^ / + ; BM-40-SPARC-GAL4 UAS-Dcr2 / +*  *w ; UAS-Tl^10B^ / + ; BM-40-SPARC-GAL4 UAS-Dcr2 / +*  *w ; vkg^G454^ UAS-myr.RFP / + ; BM-40-SPARC-GAL4 UAS-Dcr2 / +*  *w / y sc v* or *Y ; UAS-cact.RNAi^TRiP.GL00627^ / vkg^G454^ UAS-myr.RFP ; BM-40-SPARC-GAL4 UAS-Dcr2 / +*  *w / y sc v* or *Y ; UAS-cact.RNAi^TRiP.GL00627^ / vkg^G454^ UAS-myr.RFP ; BM-40-SPARC-GAL4 UAS-Dcr2 / UAS-Dif.RNAi^TRiP.HM05191^*  *w / y sc v* or *Y ; UAS-cact.RNAi^TRiP.GL00627^/ vkg^G454^ UAS-myr.RFP ; BM-40-SPARC-GAL4 UAS-Dcr2 / UAS-Rab1.RNAi^TRiP.JF02609^*  *w ; UAS-Tl^10B^ / vkg^G454^ UAS-myr.RFP ; BM-40-SPARC-GAL4 UAS-Dcr2 / +*  *w / y sc v* or *Y ; UAS-Tl^10B^ / vkg^G454^ UAS-myr.RFP ; BM-40-SPARC-GAL4 UAS-Dcr2 / UAS-Dif.RNAi ^TRiP.HM05191^*  *w / y sc v* or *Y ; UAS-Tl^10B^ / vkg^G454^ UAS-myr.RFP ; BM-40-SPARC-GAL4 UAS-Dcr2 / UAS-Rab1.RNAi^TRiP.JF02609^* |
| F | *w / y sc v* or *Y ; UAS-myr-RFP vkg^G454^ / UAS-cact.RNAi^TRiP.GL00627^ ; BM-40-SPARC-GAL4 UAS-Dcr2 / UAS-PH4αEFB.RNAi^VDRC.v2464^*  *w / y sc v* or *Y ; UAS-myr-RFP vkg^G454^ / UAS-Tl^10B^ ; BM-40-SPARC-GAL4 UAS-Dcr2 / UAS-PH4αEFB.RNAi^VDRC.v2464^* |
| G | *w ; UAS-secr.GFP / UAS-cact.RNAi^TRiP.GL00627^; BM-40-SPARC-GAL4 UAS-myr.RFP / +*  *w ; UAS-secr.GFP / UAS-Tl^10B^ ; BM-40-SPARC-GAL4 UAS-myr.RFP / +* |
| H | *w trol^CPTI-002049^ ; UAS-cact.RNAi^TRiP.GL00627^ / + ; BM-40-SPARC-GAL4 UAS-myr.RFP / +*  *w trol^CPTI-002049^ ; UAS-Tl^10B^ / +; BM-40-SPARC-GAL4 UAS-myr.RFP / +* |
| I | *y w Drs-GFP.JM804 ; BM-40-SPARC-GAL4 UAS-myr.RFP / TM6B*  *y w Drs-GFP.JM804 /* Y *; UAS-Tl^10B^ / + ; BM-40-SPARC-GAL4 UAS-myr.RFP / +*  *y w Drs-GFP.JM804 /* Y *; UAS-cact.RNAi^TRiP.GL00627^ / + ; BM-40-SPARC-GAL4 UAS-myr.RFP / +* |
| J | *y w Drs-GFP.JM804 /* Y *; UAS-Tl^10B^ / + ; BM-40-SPARC-GAL4 UAS-myr.RFP / +*  *y w Drs-GFP.JM804 /* Y *; UAS-Tl^10B^ / + ; BM-40-SPARC-GAL4 UAS-myr.RFP / UAS-Rab1.RNAi^TRiP^* *^JF02609^* |
| **Figure 7** |  |
| A | *w / Y* or *y v sc ; ppl-GAL4 UAS-myr.RFP vkg^G454^ / +; UAS-Dcr2 / UAS-shi.RNAi^TRiP.JF03133^* |
| B | *w / Y* or *y v sc ; UAS-myr.RFP vkg^G454^ / +; r4-GAL4 / UAS-shi.RNAi^TRiP.JF03133^* |
| C | *w ; UAS-shi.RNAi^NIG.18102R-1^ / + ; r4-GAL4 / +*  *w ; UAS-shi.RNAi^NIG.18102R-1^ / + ; r4-GAL4 / UAS-vkg.RNAi^NIG.16858R-3^ UAS-Cg25C.RNAi^VDRC.v28369^* |
| D | *w ; UAS-shi.RNAi^NIG.18102R-1^ / + ; r4-GAL4 / +*  *w ; UAS-shi.RNAi^NIG.18102R-1^ / + ; r4-GAL4 / UAS-vkg.RNAi^NIG.16858R-3^ UAS-Cg25C.RNAi^VDRC.v28369^*  *w ; UAS-shi.RNAi^NIG.18102R-1^ / + ; r4-GAL4 / UAS-PH4αEFB.RNAi^VDRC.v2464^*  *w; UAS-Tl^10B^ / + ; r4-GAL4 / +*  *w; UAS-Tl^10B^ / + ; r4-GAL4 / UAS-vkg. RNAi^NIG.16858R-3^ UAS-Cg25C.RNAi^VDRC.v28369^*  *w ; UAS-Tl^10B^ / + ; r4-GAL4 / UAS-PH4αEFB.RNAi^VDRC.v2464^* |
| E | *w ; puc^G462^ / TM6B*  *w ; UAS-shi.RNAi^NIG.18102R-1^ / +; BM-40-SPARC-GAL4 UAS-myr.RFP / puc^G462^*  *w ; UAS-Tl^10B^ / + ; BM-40-SPARC-GAL4 UAS-myr.RFP / puc^G462^*  *w ; UAS-cact.RNAi^TRiP.GL00627^/ +; BM-40-SPARC-GAL4 UAS-myr.RFP/ puc^G462^* |
| F | *w ; Sp / CyO ; BM-40-SPARC-GAL4 UAS-myr.RFP / TM6B*  *w ; UAS-shi.RNAi^NIG.18102R-1^ / + ; BM-40-SPARC-GAL4 UAS-myr.RFP / +*  *w ; UAS-Tl^10B^ / + ; BM-40-SPARC-GAL4 UAS-myr.RFP / +*  *w / y sc v* or *Y ; UAS-cact.RNAi^TRiP.GL00627^/ +; BM-40-SPARC-GAL4 UAS-myr.RFP/ +* |
| G | *w ; STAT10X-GFP / + ; BM-40-SPARC-GAL4 UAS-Dcr2 / +*  *w ; STAT10X-GFP / UAS-shi.RNAi^NIG.18102R-1^ ; BM-40-SPARC-GAL4 UAS-Dcr2 / +*  *w ; STAT10X-GFP/ UAS-Tl^10B^; BM-40-SPARC-GAL4 UAS-Dcr2 / +*  *w / y sc v* or *Y ; STAT 10X-GFP / UAS-cact.RNAi^TRiP.GL00627^; BM-40-SPARC-GAL4 UAS-Dcr2 / +* |
| H | *w^1118^*  *w ; UAS-shi.RNAi^NIG.18102R-1^ / vkg^G454^ UAS-myr.RFP ; BM-40-SPARC-GAL4 UAS-Dcr2 / +*  *w ; UAS-Tl^10B^ / vkg^G454^ UAS-myr.RFP ; BM-40-SPARC-GAL4 UAS-Dcr2 / +* |
| **Figure 1-figure supplement 1** |  |
| A | *w shi^ts1^ / Y ; vkg^G454^ / + ; BM-40-SPARC-GAL4 UAS-myr-RFP / +*  *shi^ts2^ / Y ; vkg^G454^ / + ; BM-40-SPARC-GAL4 UAS-myr-RFP / +* |
| B | *Canton-S*  *y w / w ; BM-40-SPARC-GAL4 / UAS-vkg.RNAi^VDRC.v16986^*  *y w / w ; BM-40-SPARC-GAL4 / UAS-Cg25C.RNAi^VDRC.v28369^* |
| C | *w / y sc v* or *Y ; vkg^G454^ UAS-myr-RFP/+ ; BM-40-SPARC-GAL4 UAS-Dcr2/ UAS-Hrs.RNAi^TRiP.JF02860^*  *w / y sc v* or *Y; vkg^G454^ UAS-myr-RFP/+ ; BM-40-SPARC-GAL4 UAS-Dcr2/ UAS-RN-tre.RNAi^TRiP.JF03085^*  *w / y sc v* or *Y ; vkg^G454^ UAS-myr-RFP/+ ; BM-40-SPARC-GAL4 UAS-Dcr2/ UAS-AP-2α.RNAi^TRiP^*^.^*^HMS00653^*  *w / y sc v* or *Y ; vkg^G454^ UAS-myr-RFP/+ ; BM-40-SPARC-GAL4 UAS-Dcr2/ UAS-AP-2μ.RNAi^TRiP.JF02875^* |
| D | *w ; vkg^G454^ UAS-myr-RFP  ; BM-40-SPARC-GAL4 UAS-Dcr2* / SM6a - TM6B  *w / y sc v* or *Y ; vkg^G454^ UAS-myr-RFP / + ; BM-40-SPARC-GAL4 UAS-Dcr2* / *UAS-shi.RNAi^TRiP.JF03133^*  *w / y sc v* or *Y ; UAS-cact.RNAi^TRiP.GL00627^/ vkg^G454^ UAS-myr-RFP; BM-40-SPARC-GAL4 UAS-Dcr2/+*  *w ; UAS-Tl^10B^ / vkg^G454^ UAS-myr-RFP ; BM-40-SPARC-GAL4 UAS-Dcr2 / +* |
| **Figure 1-figure supplement 2** |  |
|  | *y w /+ ; vkg^G454^ / +*  *y w / w ; vkg^G454^ / + ; BM-40-SPARC-GAL4 / +*  *y w / w ; vkg^G454^ / + ; BM-40-SPARC-GAL4 UAS-Dcr2 / +* |
| **Figure 2-figure supplement 1** |  |
| A | *y w / y sc v* or *Y ; BM-40-SPARC-GAL4* / *UAS-shi.RNAi^TRiP.JF03133^*  *y w / y sc v* or *Y ; UAS-Tl^10B^ / + ; BM-40-SPARC-GAL4* / *+* |
| B | *y w / y sc v* or *Y ; BM-40-SPARC-GAL4* / *UAS-shi.RNAi^TRiP.JF03133^* |
| C | *w ; Cg-GAL4 UAS-myr-RFP / UAS-Cg25C-GFP.2.1*  *w ; Cg-GAL4 UAS-myr-RFP / UAS-Cg25C-RFP.2.1*  *w^1118^* |
| D | *w ; Cg-GAL4 UAS-Cg25C-RFP.2.1/+*  *w / y sc v* or *Y ; Cg-GAL4 UAS-Cg25C-RFP.2.1/+ ; UAS-PH4αEFB.RNAi^VDRC.v2464^/+*  *w ; Cg-GAL4 UAS-Cg25C-GFP.2.1/+*  *w / y sc v* or *Y ; Cg-GAL4 UAS-Cg25C-RFP.2.1/+ ; UAS-PH4αEFB.RNAi^VDRC.v2464^/+* |
| **Figure 3-figure supplement 1** |  |
| A | *w ; Cg-GAL4 UAS-myr-RFP* |
| B | *w ; Cg-GAL4 UAS-myr-RFP*  *y w / + ; BM-40-SPARC-GAL4* / + |
| C | *y w / + ; BM-40-SPARC-GAL4* / +  *y w / y sc v* or *Y ; BM-40-SPARC-GAL4* / *UAS-shi.RNAi^TRiP.JF03133^*  *y w / y sc v* or *Y ; UAS-Rab5.RNAi^TRiP.HMC03420^ / + ; BM-40-SPARC-GAL4* / +  *y w / y sc v* or *Y ; UAS-Chc.RNAi^VDRC.v244789^ / + ; BM-40-SPARC-GAL4* / + |
| D | *y w / + ; BM-40-SPARC-GAL4* / + |
| **Figure 5-figure supplement 1** |  |
| A | *w ; vkg^G454^ UAS-myr-RFP / UAS-shi.RNAi^NIG.18102R-1^ ; r4-GAL4* / +  *w ; vkg^G454^ UAS-myr-RFP / UAS-shi. RNAi^NIG.18102R-1^; r4-GAL4* / *UAS-vkg.RNAi^NIG.16858R-3^ UAS-Cg25C.RNAi^VDRC.v28369^* |
| B | *w ; Cg-GAL4 UAS-myr-RFP / + ; 26-29-p^CA06735^ / +*  *w / y sc v* or *Y; Cg-GAL4 UAS-myr-RFP / + ; 26-29-p^CA06735^ / UAS-shi.RNAi^TRiP.JF03133^*  *w ; Cg-GAL4 UAS-myr-RFP/+ ; 26-29-p^CA06735^/ UAS-sec23. RNAi^VDRC. v24552^*  *w ; Cg-GAL4 UAS-myr-RFP/+ ; Fer1HCH^G188^/+*  *w / y sc v* or *Y; Cg-GAL4 UAS-myr-RFP/+ ; Fer1HCH^G188^/ UAS-shi.RNAi^TRiP.JF03133^*  *w ; Cg-GAL4 UAS-myr-RFP/+ ; Fer1HCH^G188^/ UAS-sec23. RNAi^VDRC. v24552^* |
| C | *w^1118^*  *w ; Fer1HCH^G188^*  *y w Drs-GFP.JM804 / Y ; UAS-Tl^10B^ / + ; BM-40-SPARC-GAL4 UAS-myr-RFP / +*  *w ; UAS-secr.GFP ; BM-40-SPARC-GAL4 UAS-myr.RFP / TM6B*  *w ; 26-29-p^CA06735^* |
| D | *w ; UAS-secr.GFP / + ; BM-40-SPARC-GAL4 UAS-myr.RFP / +* |
| **Figure 6-figure supplement 1** |  |
| A | *w ; BM-40-SPARC-GAL4 UAS-Dcr2 / TM6B*  *w / y sc v* or *Y ; UAS-cact.RNAi^TRiP.GL00627^ / +; BM-40-SPARC-GAL4 UAS-Dcr2 / +* |
| B | *w^1118^*  *cact^4^ / Df(2L)r10, cn^1^* |
| C | *w / y sc v* or *Y ; UAS-cact.RNAi^TRiP.GL00627^ / + ; BM-40-SPARC-GAL4 UAS-Dcr2 / +* |
| D | *w / y sc v* or *Y ; UAS-cact.RNAi^TRiP.GL00627^ / vkg^G454^ ; BM-40-SPARC-GAL4 UAS-Dcr2 / +*  *w / y sc v* or *Y ; UAS-Tl^10B^ / vkg^G454^ ; BM-40-SPARC-GAL4 UAS-Dcr2 / +* |
| E | *y w Drs-GFP.JM804 / + ; UAS-cact.RNAi^TRiP.GL00627^ / + ; BM-40-SPARC-GAL4 UAS-myr-RFP / +*  *y w Drs-GFP.JM804 / + ; UAS-cact.RNAi^TRiP.GL00627^ / + ; BM-40-SPARC-GAL4 UAS-myr-RFP / UAS-Dif.RNAi^TRiP TH01949.N^*  *y w Drs-GFP.JM804 / + ; UAS-cact.RNAi^TRiP.GL00627^ / + ; BM-40-SPARC-GAL4 UAS-myr-RFP / UAS-Dif.RNAi^TRiP HM05257^*  *y w Drs-GFP.JM804 / + ; UAS-cact.RNAi^TRiP.GL00627^ / + ; BM-40-SPARC-GAL4 UAS-myr-RFP / UAS-Dif.RNAi^TRiP HM05191^*  *y w Drs-GFP.JM804 / + ; UAS-Tl^10B^ / + ; BM-40-SPARC-GAL4 UAS-myr-RFP / +*  *y w Drs-GFP.JM804 / + ; UAS-Tl^10B^ / + ; BM-40-SPARC-GAL4 UAS-myr-RFP / UAS-Dif.RNAi^TRiP TH01949.N^*  *y w Drs-GFP.JM804 / + ; UAS-Tl^10B^ / + ; BM-40-SPARC-GAL4 UAS-myr-RFP / UAS-Dif.RNAi^TRiP HM05257^*  *y w Drs-GFP.JM804 / + ; UAS-Tl^10B^ / + ; BM-40-SPARC-GAL4 UAS-myr-RFP / UAS-Dif.RNAi^TRiP HM05191^* |
| F | *w ; Cg-GAL4 UAS-myr.RFP / + ; UAS-hTfR.GFP / +*  *w / y sc v* or *Y ; Cg-GAL4 UAS-myr.RFP / + ; UAS-hTfR.GFP / UAS-shi.RNAi^TRiP.JF03133^*  *w / y sc v* or *Y ; Cg-GAL4 UAS-myr.RFP / UAS-cact.RNAi^TRiP.GL00627^; UAS-hTfR.GFP / +* |
| G | *y w Drs-GFP.JM804 /* Y *; UAS-Tl^10B^ / + ; BM-40-SPARC-GAL4 UAS-myr.RFP / +* |
| H | *w ; vkg^G454^ UAS-myr.RFP / + ; BM-40-SPARC-GAL4 UAS-Dcr2 / +* |
| **Figure 8-figure supplement 1** |  |
|  | *w / y sc v* or *Y ; vkg^G454^ UAS-myr.RFP / + ; BM-40-SPARC-GAL4 UAS-Dcr2 / UAS-BM-40-SPARC.RNAi^TRiP.HMS02133^* |
