# Supplementary material for: Plasma membrane overgrowth causes fibrotic collagen accumulation and immune activation in Drosophila adipocytes
Source: eLife. 2015 Jun 19;4:e07187. doi: 10.7554/eLife.07187 (PMC4490375; doi:10.7554/eLife.07187)
Supplement: Supplementary file 3. — Origin of antibodies, mutants and transgenes used in this study. DOI: http://dx.doi.org/10.7554/eLife.07187.021 [file elife07187s003.docx]

## Supplementary File 3

Origin of antibodies, mutants and transgenes used in this study.

| **Antibodies** |  |
| --- | --- |
| anti-Cg25C | This study |
| anti-Ndg | Wolfstetter G, Shirinian M, Stute C, Grabbe C, Hummel T, et al. (2009) |
| anti-Trol | Friedrich MV, Schneider M, Timpl R, Baumgartner S (2000) |
| anti-Dorsal | Whalen AM, Steward R (1993) |
| anti-Mmp1 | Page-McCaw A, Serano J, Sante JM, Rubin GM (2003) |
| **Mutants** |  |
| *shi^ts1^* (*shi^1^*) | Kim YT, Wu CF (1990) |
| *shi^ts2^* (*shi^2^*) | Kim YT, Wu CF (1990) |
| *cact^4^* | Roth S, Hiromi Y, Godt D, Nusslein-Volhard C (1991) |
| **Transgenes** |  |
| TRIP RNAi | Ni JQ, Markstein M, Binari R, Pfeiffer B, Liu LP, et al. (2008)  Ni JQ, Zhou R, Czech B, Liu LP, Holderbaum L, et al. (2011) |
| VDRC RNAi | Dietzl G, Chen D, Schnorrer F, Su KC, Barinova Y, et al. (2007) |
| NIG RNAi | Ryu Ueda, National Institute of Genetics (Japan) |
| *vkg^G454^* | Morin X, Daneman R, Zavortink M, Chia W. (2011) |
| *UAS-myr.RFP* | Henry Chang |
| *BM-40-SPARC-GAL4* | Venken KJ, Schulze KL, Haelterman NA, Pan H, He Y, Evans-Holm M, et al. (2011) |
| *UAS-Dcr2* | Dietzl G, Chen D, Schnorrer F, Su KC, Barinova Y, et al. (2007) |
| *UAS-shi.K44A* | Moline MM, Southern C, Bejsovec A (1999) |
| *UAS-Rab5.S43N* | Entchev EV, Schwabedissen A, Gonzalez-Gaitan M (2000) |
| *UAS-Chc.DN* | Mark Muskavitch |
| *hs-Flp1.22* | Golic K., Lindquist S. (1989) |
| *act-y+-GAL4* | Ito K, Awano W, Suzuki K, Hiromi Y, Yamamoto D (1997) |
| *UAS-GFP.dsRNA* | Roignant JY, Carre C, Mugat B, Szymczak D, Lepesant JA, Antoniewski C (2003) |
| *UAS-hTfR.GFP* | Henthorn KS, Roux MS, Herrera C, Goldstein LS (2011) |
| *UAS-secr-GFP* | Pfeiffer S, Ricardo S, Manneville JB, Alexandre C, Vincent JP (2002) |
| *Cg-GAL4* | Asha H, Nagy I, Kovacs G, Stetson D, Ando I, Dearolf CR (2003) |
| *UAS-Cg25C-RFP.2.1* | This study |
| *UAS-Cg25C-GFP.2.1* | This study |
| *trol^CPTI-002049^* | Rees JS, Lowe N, Armean IM, Roote J, Johnson G, et al. (2011) |
| *r4-GAL4* | Lee G, Park JH (2004) |
| *UAS-Tl^10B^* | Jean-Marc Reichhart |
| *Drs-GFP.JM804* | Ferrandon D, Jung AC, Criqui M, Lemaitre, B, Uttenweiler-Joseph S, et al. (1998) |
| *ppl-GAL4* | Colombani J, Raisin S, Pantalacci S, Radimerski T, Montagne J, Leopold P. (2003) |
| *puc^G462^* | Morin X, Daneman R, Zavortink M, Chia W. (2011) |
| *Fer1HCH^G188^* | Morin X, Daneman R, Zavortink M, Chia W. (2011) |
| *STAT10X-GFP* | Ekas LA, Baeg GH, Flaherty MS, Ayala-Camargo A, Bach EA (2006) |
| *26-29-p^CA06735^* | Buszczak M, Paterno S, Lighthouse D, Bachman J, Planck J, et al. (2007) |
| *UAS-EGFP.shRNA.3* | Neumuller RA, Wirtz-Peitz F, Lee S, Kwon Y, Buckner M, et al. (2013) |
|  |  |
